# Supplementary material for: The locus coeruleus broadcasts prediction errors across the cortex to promote sensorimotor plasticity
Source: eLife. 2023 Jun 7;12:RP85111. doi: 10.7554/eLife.85111 (PMC10328511; doi:10.7554/eLife.85111)
Supplement: Supplementary file 1. [file elife-85111-supp1.docx]

| Figure panel | Comparison | Value compared | Mean 1 *(delta shown  for paired tests)* | SD 1 | Mean 2 *(n/a for  paired tests)* | SD 2 | Test type | P-value | N (ROIs) | N (Sites) | N (Mice) |
| --- | --- | --- | --- | --- | --- | --- | --- | --- | --- | --- | --- |
| 1D | Mean response *vs* zero | Playback halt response (stationary) [% ΔF/F] | -1.07 | 1.71 | n/a | n/a | Paired t-test | **0.0045** | n/a | 25 | 10 |
|  | Mismatch *vs* Playback halt (locomotion) | Mean response [% ΔF/F] | 2.24 | 2.30 | 0.64 | 3.51 | Rank-sum | **0.0497** | n/a | 40,24 | 13,10 |
|  | Playback halt locomotion *vs* stationary | Mean response [% ΔF/F] | 0.64 | 3.51 | -1.07 | 1.71 | Rank-sum | **0.0080** | n/a | 24,25 | 10,10 |
|  | Mean response *vs* zero | Playback halt response (locomotion) [% ΔF/F] | 0.64 | 3.51 | n/a | n/a | Paired t-test | 0.3841 | n/a | 24 | 10 |
|  | Mean response *vs* zero | Mismatch response  [% ΔF/F] | 2.24 | 2.30 | n/a | n/a | Paired  t-test | **< 10^-5^** | n/a | 40 | 13 |
|  | Mismatch *vs* Playback halt (stationary) | Mean response [% ΔF/F] | 2.24 | 2.30 | -1.07 | 1.71 | Unpaired  t-test | **< 10^-5^** | n/a | 40,25 | 13,10 |
| 1G | Mean response *vs* zero | Visual flow response (stationary) [% ΔF/F] | 3.19 | 5.31 | n/a | n/a | Signed-rank test | **0.0031** | n/a | 28 | 11 |
|  | Locomotion *vs* stationary | Mean visual response  [% ΔF/F] | -0.55 | 4.24 | 3.19 | 5.31 | Rank-sum | **0.0133** | n/a | 21,28 | 11,11 |
|  | Mean response *vs* zero | Visual flow response (locomoting) [% ΔF/F] | -0.55 | 4.24 | n/a | n/a | Paired t-test | 0.5598 | n/a | 21 | 11 |
| 2B | Posterior *vs* Anterior | Mismatch response  [% ΔF/F] | 2.56 | 2.03 | 1.18 | 2.94 | Unpaired  t-test | 0.1127 | n/a | 31, 9 | 9,4 |
|  | Posterior *vs* Anterior | Playback halt response (stationary) [% ΔF/F] | -1.11 | 1.56 | -1.00 | 2.05 | Unpaired  t-test | 0.8801 | n/a | 16, 9 | 6, 4 |
|  | Posterior *vs* Anterior | Visual flow response (stationary) [% ΔF/F] | 2.99 | 5.49 | 3.60 | 5.20 | Unpaired  t-test | 0.7855 | n/a | 19, 9 | 7, 4 |
| 2C | Posterior *vs* Anterior | Locomotion response (visual flow) [% ΔF/F] | 10.85 | 5.89 | 8.12 | 8.46 | Rank-sum | 0.3025 | n/a | 14, 6 | 6, 3 |
|  | Posterior *vs* Anterior | Locomotion response (closed loop) [% ΔF/F] | 14.99 | 7.07 | 13.33 | 7.86 | Rank-sum | 0.4257 | n/a | 28, 9 | 9,4 |
|  | Posterior *vs* Anterior | Locomotion response (no visual flow) [% ΔF/F] | 14.42 | 8.05 | 14.96 | 11.93 | Unpaired  t-test | 0.9202 | n/a | 8, 6 | 6, 3 |
|  | Posterior *vs* Anterior | Air puff response [% ΔF/F] | 24.73 | 10.03 | 24.48 | 11.23 | Unpaired  t-test | 0.9581 | n/a | 13, 8 | 6,4 |
| 3D | Mean response *vs* zero | ChrimsonR, laser response [% ΔF/F] | 0.40 | 2.69 | n/a | n/a | Bootstrap | 0.0775 | 1985 | 15 | 6 |
|  | Mean response *vs* zero | Control, laser response  [% ΔF/F] | 0.30 | 2.24 | n/a | n/a | Bootstrap | 0.1785 | 1026 | 10 | 6 |
|  | Control *vs* ChrimsonR | Laser response [% ΔF/F] | 0.30 | 2.24 | 0.40 | 2.69 | Bootstrap | 0.3929 | 1026, 1985 | 10,15 | 6,6 |
| 3E | Control *vs* ChrimsonR | Mismatch response difference [% ΔF/F] | -0.26 | 3.39 | 0.05 | 2.86 | Bootstrap | 0.2244 | 1053, 1911 | 10,14 | 6,6 |
|  | Optogenetic stimulation *vs* no stimulation | ChrimsonR, mismatch response [% ΔF/F] | 0.05 | 2.86 | n/a | n/a | Bootstrap | 0.4189 | 1911 | 14 | 6 |
| 3F | Control *vs* ChrimsonR | Visual flow response difference [% ΔF/F] | -0.04 | 2.43 | 0.06 | 2.43 | Bootstrap | 0.3852 | 1053, 2233 | 10,16 | 6,6 |
|  | Optogenetic stimulation *vs* no stimulation | ChrimsonR, visual flow response [% ΔF/F] | 0.06 | 2.34 | n/a | n/a | Bootstrap | 0.3869 | 2233 | 16 | 6 |
| 3G | Control *vs* ChrimsonR | PPE, mismatch response difference [% ΔF/F] | -0.20 | 2.97 | 0.25 | 2.93 | Bootstrap | 0.1432 | 369, 541 | 10,12 | 6,6 |
|  | Control *vs* ChrimsonR | NPE, mismatch response difference [% ΔF/F] | -0.47 | 4.41 | 0.21 | 3.81 | Bootstrap | 0.1771 | 382, 525 | 10,12 | 6,6 |
|  | Optogenetic stimulation *vs* no stimulation | ChrimsonR PPE, mismatch response [% ΔF/F] | 0.25 | 2.93 | n/a | n/a | Bootstrap | 0.2177 | 541 | 12 | 6 |
|  | Optogenetic stimulation *vs* no stimulation | ChrimsonR NPE, mismatch response [% ΔF/F] | 0.21 | 3.81 | n/a | n/a | Bootstrap | 0.3433 | 525 | 12 | 6 |
|  | Control *vs* ChrimsonR | Other, mismatch response difference [% ΔF/F] | -0.02 | 2.22 | -0.08 | 1.92 | Bootstrap | 0.5335 | 275,  526 | 10,12 | 6,6 |
|  | Optogenetic stimulation *vs* no stimulation | ChrimsonR Other, mismatch response  [% ΔF/F] | -0.08 | 1.92 | n/a | n/a | Bootstrap | 0.6313 | 526 | 12 | 6 |
| 3H | Control *vs* ChrimsonR | PPE, visual flow response difference [% ΔF/F] | 0.33 | 2.86 | 0.09 | 3.19 | Bootstrap | 0.7156 | 369, 541 | 10,12 | 6,6 |
|  | Control *vs* ChrimsonR | NPE, visual flow response difference [% ΔF/F] | -0.20 | 2.18 | -0.19 | 2.31 | Bootstrap | 0.4995 | 382, 525 | 10,12 | 6,6 |
|  | Optogenetic stimulation *vs* no stimulation | ChrimsonR PPE, visual flow response [% ΔF/F] | 0.09 | 3.19 | n/a | n/a | Bootstrap | 0.3983 | 541 | 12 | 6 |
|  | Control *vs* ChrimsonR | Other, visual flow response difference [% ΔF/F] | -0.13 | 1.79 | -0.08 | 1.49 | Bootstrap | 0.4584 | 275, 526 | 10,12 | 6,6 |
|  | Optogenetic stimulation *vs* no stimulation | ChrimsonR Other, visual flow response [% ΔF/F] | -0.08 | 1.49 | n/a | n/a | Bootstrap | 0.6316 | 526 | 12 | 6 |
|  | Optogenetic stimulation *vs* no stimulation | ChrimsonR NPE, visual flow response [% ΔF/F] | -0.19 | 2.31 | n/a | n/a | Bootstrap | 0.7564 | 525 | 12 | 6 |
| 3I | Control *vs* ChrimsonR | Locomotion modulation index | 0.22 | 0.84 | -0.15 | 0.72 | Bootstrap | **0.0012** | 816, 1687 | 8,12 | 5,6 |
| 4C | Before *vs* After | Locomotion modulation index | -0.42 | 1.00 | n/a | n/a | Bootstrap | **< 10^-5^** | 1066 | 11 | 5 |
| 4D | Before *vs* After | Locomotion modulation index | 0.04 | 1.08 | n/a | n/a | Bootstrap | 0.6271 | 1111 | 11 | 5 |
| 4E | Before *vs* After | Locomotion modulation index | 0.06 | 1.25 | n/a | n/a | Bootstrap | 0.5922 | 1140 | 10 | 5 |
| 4F | Before *vs* After | Locomotion modulation index | 0.24 | 0.98 | n/a | n/a | Bootstrap | 0.9278 | 832 | 8 | 4 |
| 4G | Before *vs* After | Visual response (locomotion) [% ΔF/F] | -0.88 | 3.54 | n/a | n/a | Bootstrap | **0.0013** | 1066 | 11 | 5 |
|  | Before *vs* After | Visual response (stationary) [% ΔF/F] | 0.17 | 2.36 | n/a | n/a | Bootstrap | 0.6929 | 1276 | 13 | 5 |
| 4H | Before *vs* After | Visual response (stationary) [% ΔF/F] | -0.17 | 2.61 | n/a | n/a | Bootstrap | 0.2579 | 1276 | 13 | 5 |
|  | Before *vs* After | Visual response (locomotion) [% ΔF/F] | -0.06 | 3.34 | n/a | n/a | Bootstrap | 0.4043 | 1111 | 11 | 5 |
| 4I | Before *vs* After | Visual response (stationary) [% ΔF/F] | -0.28 | 2.97 | n/a | n/a | Bootstrap | 0.2404 | 1140 | 10 | 5 |
|  | Before *vs* After | Visual response (locomotion) [% ΔF/F] | -0.08 | 2.61 | n/a | n/a | Bootstrap | 0.3878 | 1344 | 12 | 5 |
| 4J | Open loop *vs* Nasotemporal | Δ locomotion modulation index | 0.24 | 0.98 | -0.42 | 1.00 | Bootstrap | **0.0007** | 832, 1066 | 8, 11 | 4, 5 |
|  | Low expression *vs* Nasotemporal | Δ locomotion modulation index | 0.06 | 1.25 | -0.42 | 1.00 | Bootstrap | **0.0264** | 1140, 1066 | 10, 11 | 5, 5 |
|  | Control *vs* Nasotemporal | Δ locomotion modulation index | 0.03 | 1.01 | -0.42 | 1.00 | Bootstrap | **0.0002** | 646, 1066 | 7, 11 | 3, 5 |
|  | Temporonasal *vs* Nasotemporal | Δ locomotion modulation index | 0.04 | 1.08 | -0.42 | 1.00 | Bootstrap | **0.0014** | 1111, 1066 | 11, 11 | 5, 5 |
| 5C | CT *vs* NR | Locomotion modulation index | -0.14 | 0.92 | 0.08 | 0.87 | Bootstrap | **0.0164** | 4247, 4383 | 24,26 | 19,23 |
|  | NT *vs* CT | Locomotion modulation index | 0.08 | 0.92 | -0.14 | 0.92 | Bootstrap | **0.0308** | 1969, 4247 | 12, 24 | 8,19 |
|  | NR *vs* NT | Locomotion modulation index | 0.08 | 0.87 | 0.08 | 0.92 | Bootstrap | 0.5093 | 4383, 1969 | 26,12 | 23,8 |
| 5E | NR *vs* CT | Locomotion modulation index, fast locomotion | 0.09 | 0.88 | -0.27 | 0.90 | Bootstrap | **0.0008** | 3042, 2298 | 16,15 | 15,12 |
|  | NR *vs* CT | Locomotion modulation index, slow locomotion | 0.16 | 0.87 | -0.07 | 0.88 | Bootstrap | **0.0118** | 4669, 2907 | 31,18 | 29,16 |
|  | NR *vs* CT | Locomotion modulation index, medium locomotion | 0.10 | 0.89 | -0.14 | 0.94 | Bootstrap | **0.0178** | 4098, 3481 | 25,21 | 22,17 |
| 5G | Before *vs* After | Locomotion modulation index, fast locomotion | 0.27 | 0.97 | -0.25 | 0.92 | Bootstrap | **0.0024** | 389, 755 | 4,7 | 3,4 |
|  | Before *vs* After | Locomotion modulation index, medium locomotion | 0.35 | 0.90 | -0.03 | 0.93 | Bootstrap | **0.0429** | 872,  723 | 9,8 | 5,5 |
|  | Before *vs* After | Locomotion modulation index, slow locomotion | 0.36 | 0.85 | 0.28 | 0.91 | Bootstrap | 0.3475 | 1123, 1043 | 12,11 | 5,5 |
| 2-S1B | With *vs* without visual flow | Locomotion onset response (open loop) [% ΔF/F] | 3.03 | 7.82 | 7.17 | 6.45 | Rank-sum | **0.0442** | n/a | 20,14 | 9,9 |
| 4-S2B | Control *vs* High expression | Pupil diameter response [z-score] | -0.09 | 0.12 | 0.14 | 0.09 | Unpaired  t-test | **0.0024** | n/a | n/a | 7,6 |
|  | High expression *vs* Low expression | Pupil diameter response [z-score] | 0.14 | 0.09 | 0.00 | 0.05 | Unpaired  t-test | **0.0071** | n/a | n/a | 6,6 |
|  | Low expression *vs* Control | Pupil response [z-score] | 0.00 | 0.05 | -0.09 | 0.12 | Unpaired  T-test | 0.1242 | n/a | n/a | 6,7 |
| 4-S2D | Before *vs* After | Visual response (stationary) [% ΔF/F] | -0.70 | 3.12 | n/a | n/a | Bootstrap | **0.003** | 793 | 8 | 4 |
|  | Before *vs* After | Visual response (locomotion) [% ΔF/F] | -0.57 | 3.83 | n/a | n/a | Bootstrap | 0.0534 | 1163 | 12 | 5 |
|  | Before *vs* After | Locomotion modulation index | 0.03 | 1.01 | n/a | n/a | Bootstrap | 0.5909 | 646 | 7 | 3 |
| 4-S2E | Before *vs* After | Locomotion modulation index | 0.17 | 1.02 | n/a | n/a | Bootstrap | 0.8755 | 718 | 7 | 4 |
| 4-S2F | Before *vs* After | Visual response (stationary) [% ΔF/F] | -0.44 | 2.31 | n/a | n/a | Bootstrap | 0.1088 | 832 | 8 | 4 |
|  | Before *vs* After | Visual response (locomotion) [% ΔF/F] | -0.07 | 3.47 | n/a | n/a | Bootstrap | 0.4273 | 832 | 8 | 4 |
| 5-S1B | NR *vs* CT | Locomotion modulation index, other | 0.07 | 0.89 | -0.18 | 0.94 | Bootstrap | **0.0341** | 1276, 1384 | 23,24 | 22,19 |
|  | NR *vs* CT | Locomotion modulation index, NPE | -0.10 | 0.90 | -0.22 | 0.91 | Bootstrap | 0.1196 | 1510, 1108 | 23,24 | 22,19 |
|  | NR *vs* CT | Locomotion modulation index, PPE | 0.11 | 0.81 | -0.07 | 0.90 | Bootstrap | **0.0436** | 986, 1755 | 23,24 | 22,19 |
| 5-S1D | Before *vs* After | Locomotion modulation index, other | 0.33 | 0.90 | -0.13 | 0.93 | Bootstrap | **0.0239** | 378, 408 | 13,13 | 5,5 |
|  | Before *vs* After | Locomotion modulation index, NPE | 0.12 | 0.88 | -0.18 | 0.87 | Bootstrap | 0.0706 | 401, 418 | 13,13 | 5,5 |
|  | Before *vs* After | Locomotion modulation index, PPE | 0.51 | 0.80 | 0.25 | 0.92 | Bootstrap | 0.1006 | 372, 365 | 13,13 | 5,5 |
